# What is the functional/organic distinction actually doing in psychiatry and neurology?

**DOI:** 10.12688/wellcomeopenres.16022.1

**Published:** 2020-06-11

**Authors:** Vaughan Bell, Sam Wilkinson, Monica Greco, Callum Hendrie, Ben Mills, Quinton Deeley

**Affiliations:** 1 Research Department of Clinical, Educational and Health Psychology, University College London, London, UK; 2 South London and Maudsley NHS Foundation Trust, London, UK; 3 Department of Sociology, Philosophy and Anthropology, Exeter University, Exeter, UK; 4 Department of Sociology, Goldsmiths, University of London, London, UK; 5 Headway East London, London, UK; 6 Institute of Psychiatry, Psychology and Neuroscience, King's College London, London, UK

**Keywords:** neuropsychiatry, neurology, psychiatry, functional, organic

## Abstract

The functional-organic distinction aims to distinguish symptoms, signs, and syndromes that can be explained by diagnosable biological changes, from those that cannot. The distinction is central to clinical practice and is a key organising principle in diagnostic systems. Following a pragmatist approach that examines meaning through use, we examine how the functional-organic distinction is deployed and conceptualised in psychiatry and neurology. We note that the conceptual scope of the terms ‘functional’ and ‘organic’ varies considerably by context. Techniques for differentially diagnosing ‘functional’ and ‘organic’ diverge in the strength of evidence they produce as a necessary function of the syndrome in question. Clinicians do not agree on the meaning of the terms and report using them strategically. The distinction often relies on an implied model of ‘zero sum’ causality and encourages classification of syndromes into discrete ‘functional’ and ‘organic’ versions. Although this clearly applies in some instances, this is often in contrast to our best scientific understanding of neuropsychiatric disorders as arising from a dynamic interaction between personal, social and neuropathological factors. We also note ‘functional’ and ‘organic’ have loaded social meanings, creating the potential for social disempowerment. Given this, we argue for a better understanding of how strategic simplification and complex scientific reality limit each other in neuropsychiatric thinking. We also note that the contribution of people who experience the interaction between ‘functional’ and ‘organic’ factors has rarely informed the validity of this distinction and the dilemmas arising from it, and we highlight this as a research priority.

The functional-organic distinction attempts to differentiate symptoms, signs and syndromes that can be explained by diagnosable biological changes from those that cannot ^1^. It has been a central conceptual tool used to categorise cause and organise diagnosis in neuroscientific medicine ^2, 3^. It is cited as one of the main distinguishing characteristics of patients seen by, and referred to, psychiatrists and neurologists ^4^. It remains one of the central organising principles in current diagnostic systems, despite efforts to deemphasise the distinction in recent years ^5^.

The distinction has long been derided. In his landmark textbook of neurology, Wilson (1940) ^6^ wrote that the functional-organic distinction “lingers at the bedside and in medical literature, though it is transparently false and has been abandoned long since by all contemplative minds”. More recently it has been criticised for maintaining an artificial distinction between psychiatry and neurology ^7^, promoting naïve dualism in neuroscientific medicine ^1^, promoting diagnostic incoherence ^8^, and encouraging the continued stigmatisation of mental health problems ^9^.

Although there has been much discussion of the required conceptual basis of the functional-organic distinction, much less has been written on how it is actually used in practice. Following a pragmatist approach to conceptual analysis in psychology and medicine ^10, 11^, we examine how the functional-organic distinction has been, and is, used in medical classification, by clinicians, and in research. We use this analysis to highlight inconsistencies and contradiction. We go on to illustrate the many roles the functional-organic distinction attempts to fulfil, and then suggest how future research programmes could address some of the practical and conceptual shortcomings we identify.

## Historical shifts in the meaning of ‘functional’ and ‘organic’

Historically, the categories ‘functional’ and ‘organic’ have not retained a consistent meaning, scope, or relationship to diagnostic categorisation ^2, 3, 12^. ‘Madness’ has been considered primarily ‘organic’ or primarily ‘functional’ at different times or by different classification schemes, regardless of neurological findings ^13, 14^. Brain pathologies without structural lesions (such as seizures) have been included in both ‘functional’ and ‘organic’ categories ^2^. More recently, neuropsychiatric disorders have been interpreted in the light of cognitive science, suggesting that syndromes could be explained by impairment to distinct levels of function – either information processing (functional) or implementation (organic) ^1, 15, 16^.

Diagnostically, ‘organic’ has been used to label a specific syndrome of cognitive disturbance that explicitly excludes certain neurological disorders (as in the DSM-II diagnosis of “organic brain syndrome”) or categories of psychiatric syndromes akin to ‘functional’ diagnoses but accompanied by diagnosable neuropathology (e.g. “organic psychosis”). More recent diagnostic manuals have attempted to de-emphasise the functional-organic distinction although the changes are mostly cosmetic – by altering the terminology used to refer to ‘organic’ and changing how diagnoses are grouped. Psychiatric syndromes are now more commonly labelled as “secondary” to “disorders or diseases classified elsewhere” or “due to another medical condition” rather than ‘organic’ in both the DSM-5 and ICD although the implications are virtually identical.

## Inconsistencies in the conceptual scope of the functional-organic distinction

Although the functional-organic distinction is often cited as a tool used to differentially diagnose ‘organic’ from ‘non-organic’ disorders ^17^, the terms ‘functional’ and ‘organic’ are clearly deployed in ways that indicate more complex scope when used in practice.

‘Functional’ is often used to indicate that there is no diagnosable pathophysiology sufficient to account for the aetiology of the symptoms – as implied by the use of the term ‘functional psychiatric disorder’. However, this applies to some diagnoses and not others, despite them being identical in this regard. For example, discussion of ‘functional psychosis’ ^18^ and ‘functional depression’ ^19^ but not ‘functional autism’ or ‘functional Tourette syndrome’.

Indeed, tic disorders are diagnosed solely on behavioural characteristics, and, in fact, specifically require the exclusion of “underlying neurological disorder” (e.g. F95 Tic disorders, ICD-10) and so might be considered ‘functional’. However, ‘functional’ or ‘psychogenic’ tics are considered to be a distinct category from tics diagnosed using tic disorder criteria which are considered ‘organic’ ^20, 21^. This is also despite the existence of tic disorders that are attributed to the direct effects of neurological disorders such as traumatic brain injury ^22, 23^ and stroke ^24, 25^. As currently used, ‘organic’ tic disorder refers to the diagnosis established through the orthodox diagnostic criteria that excludes neurological damage, but also refers to tic disorder after acquired brain injury, while ‘functional’ refers to tic disorder without neurological damage but with atypical presentation and ‘psychological’ causation. Here, the conceptualisation of ‘organic’ in tic disorders covers what would otherwise be considered ‘functional’ in other disorders.

One important use of ‘functional’ is to categorise disorders that appear ‘organic’ but aren’t ^26^. For example, ‘functional neurological disorders’ are disorders that present similarly to neurological disorders but without evidence for impaired neurophysiology in the individual patient that would explain the disability, indicating their aetiology is ‘not organic’ ^27^. However, the use of ‘functional’ more broadly to signal ‘not organic’ may solely refer to diagnosable damage to the nervous system, or may also include disorders that include damage to other bodily systems. For example, the ‘functional erectile dysfunction’ indicates an erectile problem in the absence of neurological *or* vascular impairment ^28^. Here, both uses of ‘functional’ imply ‘not organic’ but the scope to which ‘organic’ refers, differs.

With the rise of ‘functional neurological disorder’ as the preferred terminology for conditions previously labelled ‘hysteria’ or ‘psychogenic’ ^29^, authors have been increasingly careful to distinguish between functional disorders, malingering and other forms of illness deception ^30^. Nevertheless, disability that presents like neurological disorder but arises without diagnosable damage to the nervous system and is not under voluntary control (‘functional neurological disorder’), is still often grouped together with the faking of symptoms under the banner of ‘functional’ syndrome or disorder ^31– 33^. Here, problems of markedly different causation, and indeed, a markedly different nature, are equally referred to as ‘functional’.

These cases illustrate that ‘functional’ and ‘organic’ are often used to indicate ‘not the other’, although the scope of the ‘other’ varies greatly depending on the context of use.

## Varying relationship to diagnostic practices that establish causality

The distinction between ‘functional’ and ‘organic’ is often treated as if the distinction is self-evident within diagnostic systems and is used as an unambiguous exclusion criteria in research (“Patients were excluded if they had an organic disorder”) and a maxim in clinical practice (“Always exclude organic causes of psychiatric symptoms”) ^34^. In practice, however, this process can be far more complex, and far more uncertain, than such statements would suggest.

David ^17^ has noted that “it is clear that the line of demarcation between organic and non-organic psychiatric disorders is not hard and fast, and in a substantial number of cases there can be continuing uncertainty” although stresses that this is not an excuse to abandon “very real distinctions between classes of disorder”. Importantly, we are not arguing here for abandoning the functional-organic distinction as entirely incoherent or futile. Indeed, there are clearly problems that unambiguously arise as a result of diagnosable biological changes, and clearly those that arise without. Nevertheless, ambiguity is probably the rule rather than exception in many practical instances of differential diagnosis.

One of the central tasks in making this distinction is attributing causality. Even when disturbed physiology is identified, clinicians then need to confidently identify it as the cause of the relevant signs or symptoms. Lishman’s criteria ^17^ suggests that organic disorders are diagnosed on the basis of “a high probability that appropriate examination and investigation will uncover some cerebral or systemic pathology responsible for, or contributing to, the mental condition”. What counts as “high probability” here remains undefined and, often, largely unexamined. In fact, the extent to which diagnosable biological changes need to be established, or causality can be confidently attributed, varies significantly between disorders as an inherent consequence of their diagnostic criteria and the investigative methods that become relevant because of them.

Delirium, a confusional state involving disturbances to cognition, behaviour and emotion, has a varying relationship to diagnosed physiological change in diagnostic manuals. In the ICD-10, “F05 Delirium, not induced by alcohol and other psychoactive substances” is an organic disorder but requires no physiological findings for confirmation. If someone fulfils the criteria for delirium (“disturbances of consciousness and attention, perception, thinking, memory, psychomotor behaviour, emotion, and the sleep-wake schedule”) they have an organic disorder by definition. The DSM-5 definition of delirium lists similar symptoms but includes the specifier that “There is evidence from the history, physical examination, or laboratory findings that the disturbance is a direct physiological consequence of another medical condition” although does not state how to establish what counts as a “direct physiological consequence”. Diagnostically, delirium is defined in a way that implies its organic nature from its presentation to the point where, in one definition, no further investigation is necessary and in another, simply stating it should be a ‘physiological consequence’ is sufficient, despite the fact that the causes of delirium are typically nonspecific and multifactorial ^35^.

In some cases, an organic basis for a disorder can be established through a hypothetico-deductive approach. For example, a patient presenting with symptoms fulfilling a DSM-5 diagnosis of panic disorder and hypercalcemia may suggest the hypothesis that the anxiety symptoms are primarily caused by hyperparathyroidism, which can cause a disturbance in blood calcium levels and increase anxiety. If the anxiety symptoms resolve or reduce after high calcium levels are treated, a diagnosis of an organic anxiety syndrome is recommended ^36^. Here a diagnosis is based on a mechanistic understanding of the pathophysiology, and an interventionist approach to hypothesis testing.

Other forms of ‘organic’ aetiology are established through apparent temporal relationship between the incident disturbance of the nervous system and the onset of psychiatric symptoms. Indeed, the psychoses of epilepsy are primarily diagnosed based on their temporal relationship to seizure events ^37^ and substance-induced psychosis is primarily diagnosed based on its temporal relationship to drug use ^38^. However, the extent to which the timing of these events can be confidently established is likely to vary due to the reliability of the informants, and the difficulty with judging the onset of psychosis itself, potentially leading to a significant role of informed speculation in the diagnostic process to help account for uncertainty.

In contrast, some organic disorders are diagnosed on a more general process of inductive inference. As Gagnon *et al.* ^39^ note, socially challenging or inappropriate behaviour is often diagnosed as organic personality disorder following a brain lesion without establishing that the particular lesion is causally responsible for the change or that personality difficulties were not present before the brain injury occurred. Evidence suggests that personality change can occur regardless of lesion location although personality change is more common in those with pre-frontal cortex lesions ^40^. However, the process of attributing the cause to a specific lesion, rendering it ‘organic personality change’, is under-determined by the presence of a lesion itself. This is particularly in light of the wide range of biopsychosocial factors that can lead to personality change after the experience of brain injury ^41^. In law, the process of attributing cause is conceptualised as the ‘but for test’ where causation is granted where the outcome would not occur ‘but for’ the injury, although even with this depth of examination, considerable ambiguity can remain ^42^. Hence, a diagnosis of ‘organic personality change’ requires a separation of ‘organic’ causes from ‘psychological’ ones, before ordering them into a hierarchy of likely importance which can only be made on a ‘most likely’ basis.

## Inconsistent use and interpretation in clinical practice

Considering there are no accepted criteria for distinguishing ‘functional’ from ‘organic’ problems across diagnoses, nor are there reliable concepts to which the terms apply across all use cases, one question is how clinicians understand the terms and concepts they regularly use. Given the importance of the functional-organic distinction for diagnosis and the prioritisation of treatment, it is perhaps surprising this has not been researched more widely. However, some existing studies have examined the question.

A mixed-methods study by Kanaan *et al.* ^26^ asked neurologists what they understood by the term ‘functional’. Survey options included “Abnormal brain function”, “Abnormal body function”, “Psychiatric problem”, and “Not organic”. The results are reproduced in Table 1 but notably all options were considered to be valid meanings of ‘functional’ by at least 20% of respondents with “not organic” being the most frequently chosen with many respondents choosing several meanings.

**Table 1.** Responses to survey question by neurologists on the meaning of ‘functional’ from Kanaan *et al.* ^26^. This table is reproduced under the terms of the Creative Common Attribution Non-commercial 2.0 (CC BY-NC 2.0) license.

*Selection*	*Proportion (%)* *choosing the selection* *at all*	*Proportion (%) of those* *choosing only that* *selection*
Abnormal brain function	127/349 (36%)	45/127 (35%)
Abnormal body function	77/349 (22%)	17/77 (22%)
Psychiatric problem	104/349 (30%)	29/104 (28%)
Not organic	216/349 (62%)	128/216 (59%)

An earlier study by Kanaan *et al.* ^43^ conducted in-depth interviews with consultant neurologists about how they understood conversion disorder – perhaps the paradigmatic functional disorder for neurologists. They endorsed psychological models of causation but didn’t feel that it was their role to derive a psychological explanation and didn’t clearly distinguish involuntary symptoms from deliberately feigning and deception under this definition.

A survey by Mace and Trimble ^44^ asked 168 British neurologists which terminology they preferred for syndromes that lack a physical explanation for the symptoms and also included a question on which syndromes should be classified as ‘functional’. The top three responses covered a remarkably wide range and included “pseudoseizures” (68%) – episodes that typically resemble tonic-clonic seizures but without accompanying seizure activity in the brain, “anxiety neurosis” (62%) – psychiatric disorders of disabling anxiety, and “Munchausen's syndrome” (61%) – a form of illness deception involving the conscious presentation of sham symptoms.

A survey of 391 Canadian psychiatrists and psychiatric residents by Benrimoh *et al.* ^45^ asked respondents to give opinions on the use of the phrase “organic causes” in their clinical work, and in psychiatry more generally. Over half of respondents (55.9%) reported they used the phrase regularly. There was considerable variation in whether the phrase was considered stigmatising, implied dualism, or led to unhelpful treatment by the medical system. Indeed, while almost 56% of psychiatrists reported using it regularly, far fewer (just under 30%) thought its use was appropriate. Many reported using it due to its assumed pragmatic function within the healthcare system, assuming, for example, that other clinicians would dismiss psychiatric patients’ reports of physical health symptoms unless they communicated ‘organic’ causation on the patients’ behalf.

Although small in number, these studies suggest that clinicians do not have a clear or consistent conceptual basis when interpreting or deploying the terms ‘functional’ and ‘organic’, despite using them frequently.

## Cultural perceptions and political uses

The functional-organic distinction has an important political dimension as attributing causes at the level of mind and body, to give a typical lay reading, or to ‘functional’ or ‘organic’, in its broader and more complex bio-medical application, imply very different things about the patient’s autonomy, responsibility and deservedness with ‘organic’ disorders seen as more deserving of care and individuals less responsible for their predicament ^46, 47^. The distinction also affects the prestige of the illness, with ‘organic’ disorders considered more prestigious than ‘functional’ disorders by both professionals and the public ^48, 49^ and with the prestige conferred on relevant medical specialities tending to reflect this same hierarchy ^50^.

The functional-organic distinction is also a basis for challenging medical authority. Challenges to the legitimacy of psychiatry have frequently suggested that valid medical specialities are necessarily identified by their focus on ‘organic’ conditions ^51, 52^ with some authors explicitly adopting the functional-organic distinction to argue against the legitimacy of psychiatric practice in the domain of ‘functional’ diagnoses ^53, 54^ although seemingly without a critical insight into the difficulties inherent in this distinction itself. Similarly, most debates over the legitimacy of syndromes included under the broad category of ‘medically unexplained symptoms’ tend to involve grassroots patient pressure to accept a largely or solely ‘organic’ explanation for the symptoms ^55^. Examples where patients lobby for non-organic explanations of controversial syndromes are far harder to come by.

## Implications

### The functional-organic distinction is unhelpfully linear and unhelpfully static

One of the notable things about the functional-organic distinction is its implied commitment to ‘zero sum’ causality ^1^. ‘Functional’ and ‘organic’ aetiology is conceived as if attributing more ‘organic’ causality necessarily implies the attribution of less ‘functional’ causality. This is apparent in the concept of “functional overlay” ^56, 57^ where a certain proportion of the total presentation is attributed to either ‘organic’ or ‘functional’ aetiology, and the wide-ranging discussion of differential diagnosis between syndromes apparently on either side of the aetiological distinction ^58– 60^. These diagnostic categories imply syndromes exist in “purely functional” or “purely organic” equivalents, presumably representing the far ends of a functional-organic spectrum.

This conceptualisation largely rejects a dynamic relationship between neurological disorder, experience, behaviour, and context. This is despite the fact that the interaction between neurocognitive capacity, perception, affect, action, and context is perhaps one of the central assumptions of the neurocognitive sciences ^61– 65^. These dynamic considerations become starker still when considering the range of difficulties likely to be analysed in terms of potential ‘functional’ and ‘organic’ components. While functions like memory may provide a relatively straightforward case (and are clearly complicated enough as they are), emotional responses and related psychiatric disorders become more complex still.

Taking depression as an example, it is very likely that the risk of depression after stroke is raised by damage to brain circuits involved in the control of emotion ^66^ and although there may be instances of post-stroke depression which are almost entirely accounted for these brain changes, the causal factors for the majority of patients are likely to include a dynamic interaction between personal, social and neuropathological factors ^67– 69^. Here, it is clear there is a marked disconnect between the best available science on the causes of depression after neurological disorder and the extent to which the functional-organic distinction can encapsulate these complex causal pathways, either through formal diagnoses or as a way of ‘apportioning causality’.

### Current diagnostic technology defines the limits of the functional-organic distinction

Another limitation of the functional-organic distinction is its reliance on clinical diagnostic technology as an arbiter of what is considered ‘organic’. Consequently, its limits lie within the extent to which this technology can detect neuropathology on an individual basis, rather than the best available science on likely causation.

In several instances, we know that damage to the nervous system is a major contributor to causality but because clinical diagnosis is unable to measure its presence, the relevant syndromes are rarely considered ‘organic’. For example, Haag *et al.*’s ^70^ review of brain injury in women subject to intimate partner violence report a prevalence of between 19% and 75% – most commonly in the form of mild traumatic brain injury where no changes can be detected on diagnostic neuroimaging. However, brain changes can be detected in group studies as altered cognition and disturbed functional connectivity ^71^. More generally, the neuropathological contributions to mild traumatic brain injury have been well-established ^72^ and the increased risk of mental health problems confidently identified ^73^. Nevertheless, the mental health consequences of intimate partner violence are almost always conceptualised in terms of social and emotional causality, with no mention of brain injury ^74, 75^. Importantly, this is not simply a matter for researchers and the development of better theoretical models. A clinician who is presented with someone who has mental health problems for which mild traumatic brain injury has been a significant causal factor will be unable to confidentially establish any ‘organic’ changes through neurological examination because such damage does not lead to neuropathology than can be currently detected on an individual basis ^76^.

It is also worth noting the reverse scenario, where clinical diagnostics regularly result in evidence for neuropathology that is often dismissed as aetiologically irrelevant despite good evidence that it is a risk factor for poor functioning and poor mental health. For example, clinically abnormal computed tomography (CT) or magnetic resonance imagining (MRI) findings are present in high proportions of individuals with first episode psychosis (64.2% ^77^; 17.6% ^78^; 19.2% ^79^). The vast majority of these findings are small but detectable pathologies, typically white matter hyperintensities, that are frequently dismissed as ‘not clinically relevant’. This is despite the fact that exactly these changes have been found to predict mental health problems, poor outcome for mental health problems ^80– 83^ and poor cognition ^84^ in otherwise neurologically unaffected adults across the lifespan.

We note psychosis is typically considered a ‘functional’ disorder, a framing which we speculate might at least partly account for why such clinical neurological findings are more likely to be dismissed in terms of explaining causality. But we also note the criterion for which abnormal findings *were* considered ‘organic’ and aetiologically relevant to psychosis in these studies ^77– 79^, namely that they were of a nature that ‘changed clinical management’ – presumably leading to a referral to neurologists for additional treatment. Here, ‘organic’ is not signifying the best evidence for likely causality but indicating a need to change clinical management.

### Social power in definition and application

Epistemic and testimonial injustice refers to the situation where a person's testimony and the credibility of their claims are questioned on the basis of negative stereotypes ^85^. Kidd and Carel ^86^ have cited ill persons as particularly likely to be subjective to testimonial injustice, due to wide-ranging stereotypes about the effect of pathologies on individuals. Neurological disorders can obviously affect the accuracy of someone’s testimony (for example, through memory deficits). However, as Kidd and Carel note, this does not change the fact that people with neurological disorders may still be subject to unjustified dismissals of valid concerns based on inaccurate ideas about personal unreliability.

We note here the significant potential for epistemic injustice given common stereotypes about ‘functional’ and ‘organic’ illnesses in terms of autonomy, responsibility and deservedness ^46^. Research on carer and professional perceptions of ‘challenging’ behaviour in survivors of brain injury show clear evidence for the active construction of the causes of behaviour ^87, 88^. Here, the extent to which the person’s troubling behaviour is given a ‘brain injury’ or ‘intentional’ explanation depends heavily on the motivations of the individual doing the interpretation. Huet *et al.* ^89^ reported exactly this process of active interpretation by health professionals who tended to reframe aggressive and angry behaviour as involuntary, thereby maintaining a ‘good person’ understanding of the patient. However, this interpretation also has the potential to erase any valid frustrations or concerns that may have motivated the behaviour and renders the individual socially inert.

Although not widely researched, we note that the concept of ‘inappropriate’ or ‘challenging’ behaviour relies heavily on social and cultural norms and has the potential to raise important ethical issues. Cases of changes in sexual preference and sexual orientation after brain injury have been interpreted in terms of pathological alteration to the brain circuits mediating sexual preference ^90^. But it is also possible that the brain injury altered the capacity to strategically inhibit pre-existing desires, or that the change was a conscious decision after an important life event, although these latter interpretations require a starker form of social attribution that may involve re-evaluating, rightly or wrongly, the person in question, depending on others’ approval of their new behaviour. We note that changes subject to fewer prejudices and typically seen in more benign light, such as a sudden interest in producing art after brain injury, are usually explained in terms of ‘disinhibition’ ^91, 92^ which has the function of attributing the new socially acceptable activity to the ‘self’ rather than to pathology, which has simply ‘released’ it.

Common to these accounts is that the testimony of patients features little in the explanation of the behaviour and we suggest that this situation occurs frequently in the process of providing both clinical accounts and scientific explanations. Furthermore, we also note that the testimony of people affected by neurological and neuropsychiatric conditions is almost entirely absent from the scientific and clinical debates that have formed the conceptual basis of the functional-organic distinction. Here, we argue that inclusion of first-person perspectives is essential to inform several important areas of practice and scientific understanding.

Firstly, it would inform clinical work in terms of better understanding the process of being subject to the functional-organic distinction, how it is perceived, experienced and understood by patients. Secondly, in terms of scientific understanding, it would provide a phenomenology of experience to better understand the interaction between, for example, injury and autonomy. These approaches are now commonplace in psychiatry, where understanding experience is considered to be a central component in advancing the development and delivery of health care systems ^93^ and where understanding subjective experience informs neuropsychological theories of causation ^94^. Although some studies have been conducted on the experience of health care, as far as we know, no research has ever been conducted with, for example, survivors of brain injury that aims to inform the science of how neurological-level and personal-level processes interact.

It is also the case that the priorities of people who use healthcare systems may differ markedly from the priorities of healthcare systems themselves. Similarly, the research priorities of researchers and patients have been found to differ substantially ^95^. We note here that the functional-organic distinction is a conceptual tool developed by medicine to try and solve a particular set of problems, but one important focus of research should be to investigate how well these problems actually map onto the priorities of those seeking help.

## Conclusions

Before tackling the question of what the functional-organic distinction is doing in psychiatry and neurology, it is perhaps worth noting what it is not doing. It is not reliably distinguishing between aetiology at different levels (physiological, psychological etc) across contexts. Indeed, the extent to which it can reliably distinguish between types of causes for particular signs, symptoms and syndromes seems to differ depending on the signs, symptoms and syndromes being assessed. In some cases, conceptual inconsistencies and difficulties with practical diagnosis render this an ambition rather than a reliable outcome, partly due to the multiplicity of meanings represented by the terms themselves. Rather than a general distinction, it is more akin to various local distinctions, each defined and limited by context.

Importantly, it seems that one of the major functions of this distinction is to provide a justification and language to allow clinicians to prioritise healthcare interventions. Indeed, considering the complex nature of neuropsychiatric disorders where causes are likely dynamic, reciprocal and span levels of explanation, the functional-organic distinction often seems like a tool that helps determine treatment priority dressed up in the language of causation. To reiterate, it is clear that there are syndromes almost entirely accounted for by diagnosable pathophysiological changes, and those that are not, but most neuropsychiatric disorders are not at these extremes, and are caused by multiple interacting factors.

Rhetorically, however, talk of ‘organic’ causation retains a caché, influence and credibility that ‘functional’ causation does not, and it is clear that this rhetoric is used strategically by healthcare professionals to work within healthcare systems – mostly, it must be said, in good faith attempts to provide effective care. Nevertheless, the extent to which the strategic simplification and complex scientific reality influence and limit each other should be more widely investigated.

Perhaps most striking is the fact that these debates almost entirely exclude the priorities and experiences of those most affected by them – namely patients with challenges of mind, brain, emotion, behaviour, and society, whose difficulties are interpreted in terms of ‘functional’ and ‘organic’ components. We highlight how the inclusion of these perspectives are likely to be essential for better science and better healthcare in this area.

## Data availability

### Underlying data

No data are associated with this article.
