# Supplementary material for: What is the functional/organic distinction actually doing in psychiatry and neurology?
Source: Wellcome Open Res. 2020 Jun 11;5:138. [Version 1] doi: 10.12688/wellcomeopenres.16022.1 (PMC7338913; doi:10.12688/wellcomeopenres.16022.1)
Supplement: Peer review history

# Peer review: Reviewer response for version 1

**DOI:** 10.21956/wellcomeopenres.17576.r39089

**Published:** 2020-07-21

**Authors:** Ana Gómez-Carrillo

**Affiliations:** 1 Division of Social and Transcultural Psychiatry, McGill University, Montreal, QC, Canada

This incisive narrative review by Bell *et al.* provides an overview of the tensions – representatively captured in ongoing use of the organic/functional distinction — that likely divide(d) and continue to plague the fields of psychiatry and neurology.

 The authors do an excellent job at reviewing a complex issue with a broad scope and bring imperative concerns to bear that research and practice must re-visit. In so doing it raises more questions than it answers. While serving to illustrate the complex scope of the distinction in practice the pragmatist approach falls short of considering deeper and earlier roots of the distinction further afield.

 The review is timely in that it points out how clinical practice is stuck in paradigms that have not caught up with more contemporary theories in cognitive sciences central to psychiatry and neurology. Although hinted at in the abstract and in the section on implications (“the dynamic relationship between cognitive capacity, affect, perception, action and context”) the pragmatist approach taken leaves this divide between research and practice insufficiently addressed – as such this important discussion feels slightly stuck, seemingly engaging with older conceptions and less so with more contemporary ones. Perhaps this is simply a reflection of the slow uptake and resistance to change that characterizes clinical practice which is what the authors chose to focus on here.

 By exposing the inconsistencies and contradictions of the uses of the f/o distinction the authors clearly question the usefulness and validity of this distinction and expose the dichotomies that it perpetuates. Beyond what the distinction is or is not doing – is the question of why it is still there (conceptually and causally)? There seems to be some value to this distinction, some functions or needs that it fulfills which apparently outweigh its conceptual (and pragmatic) incoherencies. In referring to Wilson’s quote on whether more contemporary understandings of the brain have perhaps left this distinction irrelevant the authors approach this issue but take it no further. A more explicit positioning or discussion on whether and, if so, how more contemporary theories may or may not be an alternative would strengthen the manuscript.

 In what follows I share some suggestions that may help add clarity in structure and argumentation as well as reflections to further this discussion:

- When reviewing historical shifts in the meaning of f/o, briefly linking the distinction to its conceptual origins would help untangle and frame some of the contradictions the authors eloquently exemplify. I was surprised to find no reference to the psychodynamic literature that largely shaped the use of “functional” as it relates to conversion disorders and variations thereof, including historical links to the polymorphous concept of hysteria. Aware that the literature on this is extensive and controversial as well as perhaps not central to this review – much of the uses of the ‘functional’ classifier across disciplines rely explicit or implicitly on psychodynamic theories. 

- Also, briefly characterizing ‘dualism’ in a bit more detail and how it relates to the f/o distinction would serve to set the stage for the examples and analysis that follow e.g. substance dualism vs epistemic dualism or how the distinction relates to amoral naturalistic and moral super-naturalistic domains.

- As the authors note, the use of this distinction varies significantly by context and although they provide a thorough overview of how the f/o distinction is used across contexts attempting to review its use in both psychiatry and neurology and research and practice at once poses a challenge. At times this comes at the cost of glossing over more necessary differentiations and risks producing an overview that is more complex than the use of this distinction already is. A clearer picture may emerge if a more systematic approach based on when, where and by whom the distinction is being applied structured the examples provided.

- For example, psychosis is noted to be typically considered functional – from whose perspective? Interestingly, while from a neurologist perspective the f/o distinction may draw the boundaries of neurology and psychiatry, artificially or not, mainstream psychiatry currently continues to prioritize organic etiological understandings for its ‘classic’ diagnoses despite lacking the necessary evidence for now.

- Indeed, the organic-functional distinction is intimately related to and perhaps even sustained by issues that underlie the stigma of mental disorders. But does the stigmatization stem from this distinction– or does this distinction merely reinforce it? Stigma and the f/o distinction share origins and possibly persist in one form or another globally because of an implicit ontology that emerges from a residual form of mind-body dualism **. **If so is it a matter of ridding ourselves from this distinction?

- Regarding the inconsistent use of the f/o distinction, differentiating between the use of this terminology formally and its use as part of medical jargon across medical specialties (beyond its use for ‘turfing’) would lead to a clearer picture in the discussion and the examples provided. The authors seem to allude to this in saying that clinicians use the distinction strategically. But even the use of this distinction in medical jargon varies significantly across specialties, their agenda’s and who is being communicated with – so while generalizing allows to make the point, it may fail to capture the nuance needed to advance this analysis. Functional is used in some contexts as a pseudo-euphemism for psychosomatic (as if to avoid stigma related reactions), others use the terms interchangeably and some make a distinction. The authors may consider thinking one of the examples of the f/o distinction in detail through to the end including the multiple explanatory models and tensions it captures as well as the specific clinical niche and cultural practice it originates in. This would expose the potential imperatives that lead to the use of this distinction as well as the pitfalls. In the example of delirium, despite its multifactorial etiology, it is a clinical imperative for clinicians to consider delirium a consequence of an underlying medical disorder or physiological imbalance – why is this so? What would be the consequences of not making the distinction here? The example serves to convey the complexity of this distinction however a straightforward pragmatic clinical predicament underlies the issue here. The examples provided in the manuscript are very good and I am not suggesting others should necessarily be added.

- While in some cases the uses of the f/o distinction in multifactorial pathologies relate more to clinical management of ambiguity and uncertainty e.g. delirium requiring a binary clinical decision tree, other uses are better explained by the forms of ambiguity and uncertainty that underlie cultural ascriptions and understandings of agency, moral and responsibility e.g. organic personality change. It is always some degree of both. The authors rightly highlight how the terminology fails to cover the pragmatic and philosophical function of this use but no alternative is discussed. The extent to which the tensions uncovered are bound to management of ambiguity and uncertainty in both clinical practice and a person’s illness experiences remains to be discussed and the insights of people who experience the interaction between function and organic will be of great value here. Ambiguity lends itself to be exploited to project one’s own intentions, motivations, understandings, models and needs. The ambiguity will likely remain and be shaped by individuals’ needs and perspectives again and again. Overcoming these tensions will require better training in and integration of complex thinking on one level, and alternate ways to reduce complexity to the binary choices that permit action on another. This is easier in theory and discourse but harder when faced with the pragmatic reality of life, clinical decision making, insurance claims or court rooms…

This is a worthy discussion with impact for people who experience the interaction between function and organic and clinicians alike. The points above may help look beyond the conundrum of this distinction to see if the issues that are linked to it are deeper rooted and prone to prevail until a more widespread social paradigm change is achieved, independently of changes to nomenclature or advances in theoretical understandings. Can the f/o distinction be viewed as other than two complementary aspects of illness and disease in line with Bohr’s notion of complementarity? The contribution of people who experience the interaction between function and organic will be central here – more than to clarify its use or validity, to unpack the origin of the problems this distinction has tried to solve and help advance better alternatives. I look forward to more work on this topic by the group and to the conclusions they may reach.

Is the review written in accessible language?

Yes

Are all factual statements correct and adequately supported by citations?

Yes

Are the conclusions drawn appropriate in the context of the current research literature?

Yes

Is the topic of the review discussed comprehensively in the context of the current literature?

Partly

Reviewer Expertise:

transcultural psychiatry, philosophy and psychiatry, mood disorders

I confirm that I have read this submission and believe that I have an appropriate level of expertise to confirm that it is of an acceptable scientific standard.

# Peer review: Reviewer response for version 1

**DOI:** 10.21956/wellcomeopenres.17576.r39086

**Published:** 2020-07-06

**Authors:** Jesús Ramirez-Bermudez

**Affiliations:** 1 Neuropsychiatry Unit, National Institute of Neurology and Neurosurgery of Mexico, Mexico City, Mexico; 2 School of Medicine, National Autonomous University of Mexico, Mexico City, Mexico

This is a necessary scientific essay dealing with the conceptual duality between “the organic” and “the functional” as analitic categories in clinical practice. Opening review and the definitions provided by the authors are useful and I would not recommend further bibliography regarding this part. Also, the critique is well developed, although I think the authors should discuss the alternatives to this conceptual duality in clinical practice. A few notes:

1. The paragraphs dedicated to *Historical shifts in the meaning of ‘functional’ and ‘organic’* are fine and I have no further comments.
2. Regarding the paragraphs dedicated to *Inconsistencies in the conceptual scope of the functional-organic distinction *and *Varying relationship to diagnostic practices that establish causality*: I agree with the authors’ discussion, although some other clinical problems merit consideration:
  1. First, the case of migraine and other disorders which are considered “neurological” and are attended by neurologists, although the clinical diagnosis is not based in biological markers; the scientific epistemology and the clinical approach to patients with migraine is similar to the epistemology/approach which is used in psychiatric patients (a diagnosis based in symptoms by means of clinical interviews, etc). However, this clinical problems are not grouped traditionally with the “functional” disorders nor are dismissed as “psychological”.
  2. Second: Parkinson’s Disease, among other neurological diagnoses, is interesting also for the discussion, because in clinical practice, rarely the diagnosis is based in technological procedures. Some difficult cases may be solved with support of PET, but the significant majority of cases are diagnosed by means of the identification of a clinical phenotype which is captured by means of the highly trained visual and tactile abilities of the clinician, and some times with support of clinical scales and pharmacologic trials (UPDRS, l-dopa test). Although the neuropathology of Parkinson’s Disease is well stablished, the recognition of the disease in clinical practice is based in most cases purely by clinical skills, and other resources (brain imaging, etc) are used to rule out other etiologies which may produce similar phenotypes. Certainly, Parkinson’s Disease is considered organic. Clinicians may use similar approaches in field of dementias, although in this case the technological devices have a more prominent role. In our center, MRI and PET scans are quite relevant in the diagnosis of Alzheimer’s Disease, Frontotemporal Dementia and so on, but those diagnosis are also made with less emphasis in technology in different settings, and the logic of this issue is the same: the recognition of the clinical phenotype is enough to classify the patient as having an “organic” disorder. The authors pose a similar problem regarding the “delirium” diagnosis.
  3. I include this discussion related to migraine, Parkinson’s Disease and the Dementias as it serves me to pose some general questions, although I don’t think these diagnoses should be necessarily included in the manuscript.
3. The paragraphs related to *Inconsistent use and interpretation in clinical practice *and *Cultural perceptions and political uses* are fine from my perspective.
4. Regarding the *Implications* section, I only have two comments:
  1. I totally agree with the paragraphs related to Social power in definition and application, but it is not clear to me how this is particularly relevant to the discussion between “Organic vs Functional”. Or is it a general issue related to psychiatric stigma?
  2. I totally agree that the utility and the scientific value of the “Organic vs Functional” distinction is limited, to say the least. However, I wonder if the authors can discuss more the alternatives. In the Perminder Sachdev paper, some of the alternative terms and views are discussed, including concepts as “primary” “secondary” “symptomatic” “idiopathic”. This terminologies, along with their conceptual implications, are relevant from my point of view to the discussion. Although I understand that the authors are not arguing simply for a change in terminologies and go on to discuss the validity of the dichotomic perspectives, it would be of great value if they can advance at some degree their alternative proposals. Perhaps this could be related to the following paragraph: “No research has ever been conducted with, for example, survivors of brain injury that aims to inform the science of how neurological-level and personal-level processes interact.” The Sachdev paper discusses practical approaches beyond terminology that go to the attribution of causality.
  3. Perhaps a general discussion of what clinical-epidemiological sciences tell us of the attribution of causality in groups of patients is important, as a background reflection. Myself, I find a great approach in the Elwood’s textbook: Critical Appraisal of Epidemiological Studies and Clinical Trials. However, a different question is how clinicians establish causality at the individual level? This question is quite relevant along the neurological-psychiatric spectrum of problems and is probably overlooked in the scientific & philosophic literature.

Is the review written in accessible language?

Yes

Are all factual statements correct and adequately supported by citations?

Yes

Are the conclusions drawn appropriate in the context of the current research literature?

Yes

Is the topic of the review discussed comprehensively in the context of the current literature?

Yes

Reviewer Expertise:

Clinical neuropsychiatry

I confirm that I have read this submission and believe that I have an appropriate level of expertise to confirm that it is of an acceptable scientific standard.

# Peer review: Reviewer response for version 1

**DOI:** 10.21956/wellcomeopenres.17576.r39090

**Published:** 2020-06-25

**Authors:** Norman A. Poole

**Affiliations:** 1 Department of Neuropsychiatry, St George's Hospital, South West London and St George's Mental Health NHS Trust, London, UK

Bell *et al*'s paper is a useful and thoughtful reappraisal of the widely used but under examined functional vs organic distinction in neurology and psychiatry. In particular, it draws attention to the incoherence of the distinction, which differs in content across its various applications and they illustrate the point with well observed examples such as differentiating between organic and functional tics. 

 Given the longstanding conceptual incoherence of the distinction - they approve Wilson's skewering of it in his 1940's textbook - why then does it endure in clinical practice and research? The evidence they cite addressing this is limited but telling. Neurologists are prone to equate functional with feigned or demarcating a psychological terrain not to be explored while psychiatrists employ organic when they fear their patient's symptoms will be ignored by medical colleagues. This functional - that word again! - analysis  seems to me to approach the nub of the issue and overlaps with but is not quite the same as the question of aetiology.

 It is probably a reification of the dichotomy to believe that it does any real work differentiating biomedical from psychosocial causality and the biopsychosocial model can be applied to most conditions doctors treat. In clinical practice the terms are largely used where there is a dispute about which specialism should take the lead in the patient's care. What is being communicated when neurologists say 'functional' and psychiatrists invoke 'organic' is really one saying to the other, 'Over to you.' This can be read as a base  abdication of clinical responsibility or an act of deference to the appropriate specialism, and in truth is probably a bit of both.

 It has become a truism that organic conditions and their attendants are somehow superior in the hierarchy of disease and this narrative review falls prey to the same assumption. The organic/functional dichotomy maps neatly on to folk theorising about mental disorder; lay people quite naturally understand mental symptoms to be biologically or psychologically caused with the one negating the other ^1^. However, the assumptions that flow from this turn out to be unreliable. Biological explanations are in fact more stigmatising and fatalistic than those that utilise psychological concepts ^2^.

 This tendency to dichotomise ought to be challenged though, as it does not conform to current scientific knowledge while fostering therapeutic nihilism, and this paper marks a significant attempt to do just that. It accords moves elsewhere in the literature. After facing some sustained criticism the concerns about vagueness ^3^ and incompatible levels ^4^ within the biopsychosocial model of disease are being addressed. Doctors must recognise and communicate aetiological complexity to protect themselves and their patients from falling into simplistic, but seemingly inherent, patterns of thought.

 Yet I was left slightly confused as to whether the authors are rejecting the distinction outright, or not. And if so what would it be replaced with? I am in full agreement that it fails to carve nature at her joints aetiologically, but perhaps less convinced that this is its real purpose. The clinical use of 'functional' exposes biomedicine's abhorrence of the 'other' while 'organic' is psychiatry's codeword for medics to take the patient seriously. Sadly, it will take more than a new set of terms for that to change.

Is the review written in accessible language?

Yes

Are all factual statements correct and adequately supported by citations?

Yes

Are the conclusions drawn appropriate in the context of the current research literature?

Yes

Is the topic of the review discussed comprehensively in the context of the current literature?

Yes

Reviewer Expertise:

Neuropsychiatry, Philosophy and psychiatry, Psychopathology

I confirm that I have read this submission and believe that I have an appropriate level of expertise to confirm that it is of an acceptable scientific standard.

## References

[1] : Psychiatric categories as natural kinds: Essentialist thinking about mental disorder. *https://www.researchgate.net/publication/286866478_Psychiatric_categories_as_natural_kinds_Essentialist_thinking_about_mental_disorder*.2000;

[2] Natural Kinds in Psychiatry: Conceptually Implausible, Empirically Questionable, and Stigmatizing.2014; 10.7551/mitpress/8942.003.0003 10.7551/mitpress/8942.003.0003

[3] : The Biopsychosocial Model of Health and Disease New Philosophical and Scientific Developments. *Palgrave Macmillan*.2019;31886965

[4] : Levels of analysis in psychopathology: cross-disciplinary perspectives. *Cambridge University Press*.2020;
